# Theophylline inhibits the cough reflex through a novel mechanism of action^☆^

**DOI:** 10.1016/j.jaci.2013.11.017

**Published:** 2014-06

**Authors:** Eric Dubuis, Michael A. Wortley, Megan S. Grace, Sarah A. Maher, John J. Adcock, Mark A. Birrell, Maria G. Belvisi

**Affiliations:** Respiratory Pharmacology, Pharmacology & Toxicology, National Heart and Lung Institute, Faculty of Medicine, Imperial College London, London, United Kingdom

**Keywords:** Sensory nerves, vagus, cough, ion channels, capsaicin, BK_ca_ channel, Large-conductance calcium-activated potassium channel, [Ca^2+^]_i_, Intracellular calcium, COPD, Chronic obstructive pulmonary disease, DiI, DilC18(3)-1,1′-dioctacetyl-3,3,3′,3′-tetramethyl-indocarbocyanine perchlorate, ECS, Extracellular solution, IK channel, Intermediate-conductance calcium-activated potassium channel, PDE, Phosphodiesterase, PGE_2_, Prostaglandin E_2_, SK channel, Small-conductance calcium-activated potassium channel, TRP, Transient receptor potential

## Abstract

**Background:**

Theophylline has been used in the treatment of asthma and chronic obstructive pulmonary disease for more than 80 years. In addition to bronchodilator and anti-inflammatory activity, clinical studies have suggested that theophylline acts as an antitussive agent. Cough is the most frequent reason for consultation with a family doctor, and treatment options are limited. Determining how theophylline inhibits cough might lead to the development of optimized compounds.

**Objective:**

We sought to investigate the inhibitory activity of theophylline on vagal sensory nerve activity and the cough reflex.

**Methods:**

Using a range of techniques, we investigated the effect of theophylline on human and guinea pig vagal sensory nerve activity *in vitro* and on the cough reflex in guinea pig challenge models.

**Results:**

Theophylline was antitussive in a guinea pig model, inhibited activation of single C-fiber afferents *in vivo* and depolarization of human and guinea pig vagus *in vitro*, and inhibited calcium influx in airway-specific neurons *in vitro*. A sequence of pharmacological studies on the isolated vagus and patch clamp and single-channel inside-out experiments showed that the effect of theophylline was due to an increase in the open probability of calcium-activated potassium channels. Finally, we demonstrated the antitussive activity of theophylline in a cigarette smoke exposure model that exhibited enhanced tussive responses to capsaicin.

**Conclusion:**

Theophylline inhibits capsaicin-induced cough under both normal and “disease” conditions by decreasing the excitability of sensory nerves through activation of small- and intermediate-conductance calcium-activated potassium channels. These findings could lead to the development of optimized antitussive compounds with a reduced side effect potential.

Theophylline has been used in the treatment of respiratory diseases, such as asthma and chronic obstructive pulmonary disease (COPD), for more than 80 years. Although in industrialized countries β-agonists and steroids are the preferred treatment, globally, theophylline remains a widely prescribed drug, particularly in patients with more severe disease, because it is cheap and readily available.^1^ Although it has been primarily prescribed for its bronchodilator activity, there is a large body of evidence suggesting that it also possesses anti-inflammatory activity in both asthmatic patients and patients with COPD.^2-4^ Furthermore, clinical studies have shown that adding theophylline to inhaled steroids in patients with mild-to-moderate asthma and to long-acting β-adrenoceptor agonists in patients with severe COPD provides additional clinical improvement. In addition to these properties, preclinical and clinical studies have suggested that both theophylline and another methylxanthine, theobromine, act as antitussive agents in several preclinical studies and in patients with a range of clinical conditions.^5,6^ In children and adults with poorly controlled asthma, theophylline significantly improved symptom scores for cough and wheeze compared with placebo.^7^ Theophylline has also been recommended for the treatment of cough in patients with COPD^8^ and has been shown to be effective for treating angiotensin-converting enzyme inhibitor–related cough.^9^

Cough is a protective reflex and defense mechanism in healthy subjects.^10^ However, cough is also the most common respiratory complaint for which medical attention is sought,^11^ often presents as the first and most persistent symptom of many respiratory diseases, and can be idiopathic in nature.^12^ Chronic persistent cough has a profoundly detrimental and debilitating effect on quality of life that can lead to social isolation and clinical depression.^13^ Cough is a major problem that leads patients to use over-the-counter remedies as first-line treatments; United Kingdom sales were greater than £350 million in 2004, and US sales were greater than $2 billion.^14^ However, a recent meta-analysis established that there was no good evidence for the effectiveness of such remedies.^15^ Therefore the mechanisms underlying chronic cough and the identification of novel therapeutic targets for its treatment present a grossly neglected and unmet clinical need.

In this study we have demonstrated that theophylline inhibits sensory nerve activation and the cough reflex and uncovered a possible mechanism of action. This study highlights a previously unrecognized benefit of theophylline that could explain the positive effects seen in patients with respiratory disease. Elucidating the mechanism of action could lead to the development of optimized compounds with a reduced side effect profile.

## Methods

For a more detailed discussion of the methods used in this study, please see the Methods section in this article's Online Repository at www.jacionline.org.

### Animals

Male guinea pigs (Dunkin-Hartley, Harlan, United Kingdom) weighing 350 to 450 g (400-750 g for single-fiber *in vivo* studies) were housed in temperature-controlled (21°C) facilities with food and water *ad libitum* for at least 1 week before the experiment. All experiments were performed in accordance with the UK Home Office guidelines for animal welfare based on the Animals (Scientific Procedures) Act of 1986.

### Effect of theophylline on citric acid–and capsaicin-evoked cough in conscious guinea pigs

Conscious unrestrained guinea pigs were placed in individual plastic, transparent, whole-body plethysmograph chambers, and cough was detected, as previously described.^16,17^

### Effect of theophylline on capsaicin-induced firing of single-fiber afferents and bronchospasm *in vivo*

Guinea pigs were anaesthetized with urethane (1.5 g/kg) intraperitoneally. The trachea was cannulated, and pressure was measured with an air pressure transducer connected to a side arm of the tracheal cannula. Animals were paralyzed with vecuronium bromide, initially administered at a dose of 0.10 mg/kg intravenously, followed every 20 minutes with 0.05 mg/kg administered intravenously to maintain paralysis. Firing of single-fiber afferents and bronchospasm was measured, as previously described.^18^

### Effect of theophylline on depolarization of the vagus nerve preparation to various tussive agents

Guinea pigs were culled with an overdose of pentobarbitone (200 mg/kg administered intraperitoneally). The 2 vagus trunks were carefully dissected free and placed in Krebs–Henseleit solution. Segments of the vagus nerve were mounted in a “grease-gap” dual recording chamber system, as previously described.^19,20^

### Primary culture of sensory neurons

Calcium imaging of primary sensory jugular ganglia was performed, as previously described.^21^ Identification of airway sensory neurons was done by means of intratracheal administration of the retrograde neuronal tracer DilC18(3)-(1,1′-dioctacetyl-3,3,3′,3′-tetramethyl-indocarbocyanine perchlorate) (DiI; Invitrogen, Carlsbad, Calif) 2 weeks before collecting the ganglia and isolating the cells. Camptothecin was used to inhibit mitotic cell growth during the primary culture of neurons, and airway neuron staining with DiI was assessed with 531/40 nm and 593/40 nm excitation/emission filters (BS 565) before experimentation. Intracellular calcium [Ca^2+^]_i_ and membrane voltage changes were simultaneously optically measured in primary cultured dissociated jugular neurons (see the Methods section in this article's Online Repository). The effect of theophylline on small-conductance calcium-activated potassium channel (SK channel) and intermediate-conductance calcium-activated potassium channel (IK channel) currents and analysis of the direct effect of theophylline on calcium-activated potassium channels in the jugular ganglia were also investigated (see the Methods section in this article's Online Repository).

### Cigarette smoke exposures

Guinea pigs were exposed to cigarette smoke from research cigarettes (3R4F, with filters removed; University of Kentucky, Lexington, Ky) for 1 hour twice daily, with 4 hours between each bidaily exposure period. Cigarette smoke exposure protocols are described in the Methods section in this article's Online Repository and were similar to those described by Eltom et al.^22^

### Drugs and solutions

All compounds and drugs used are described in the Methods section in this article's Online Repository. The amount of vehicle (dimethyl sulfoxide) was limited to 0.1% to avoid possible side effects, and the effect of theophylline at each concentration was obtained in the presence of the same amount of vehicle each time. In all the experimental design paradigms, the effect of vehicle alone was assessed, and no significant effect observed.

## Results

### Effect of theophylline in a conscious guinea pig cough model

**Fig 1:**
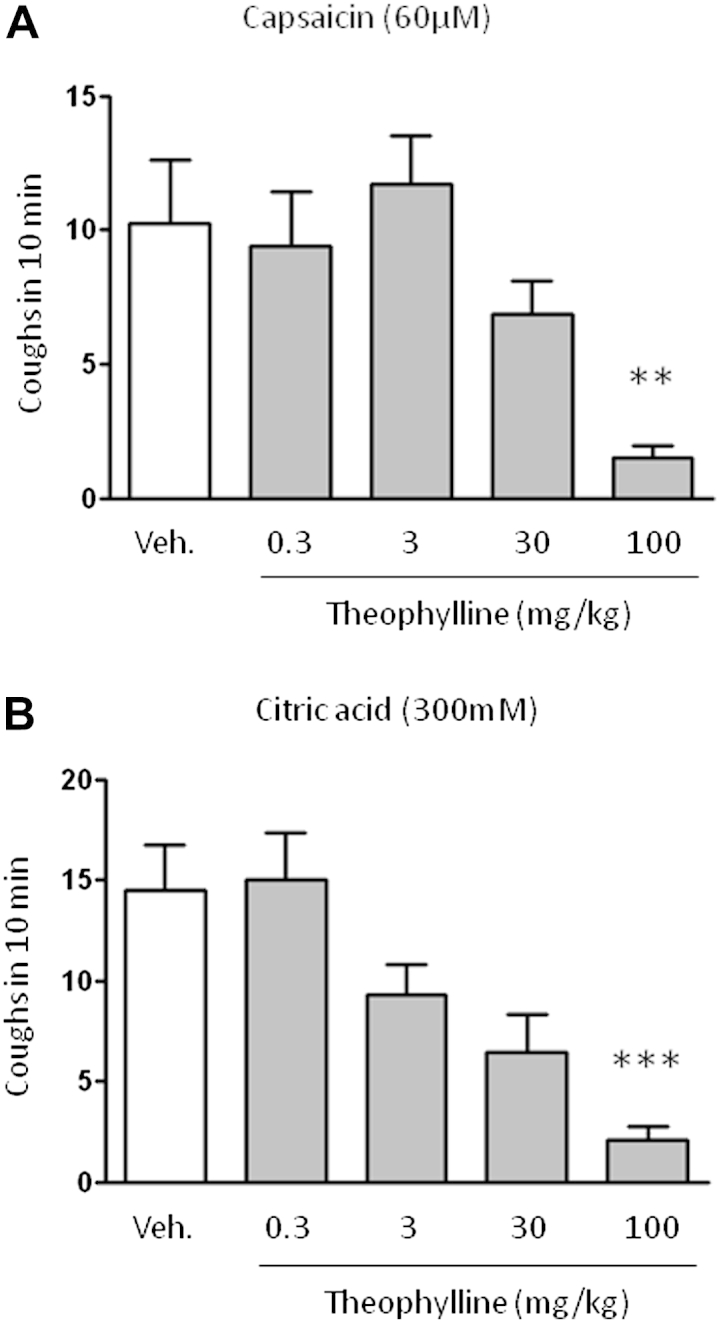
Effect of theophylline on induced cough in a conscious guinea pig model. The effect of theophylline on the number of coughs induced by nebulized solutions of capsaicin (5 minutes, n = 15; **A**) or citric acid (10 minutes, n = 12-15; **B**) into the plethysmograph recording chamber and recorded over 10 minutes from the start of nebulization is shown. The vehicle (*Veh*; 0.5% methylcellulose and 0.2% Tween 80 in saline administered intraperitoneally) or theophylline (administered intraperitoneally) dose 1 hour before cough challenge is indicated. Data are presented as means ± SEs. **P < .01 and ***P < .001 using 1-way ANOVA with the Dunnett multiple comparison *post hoc* test.

Theophylline (1 hour before treatment) decreased the number of coughs induced by aerosolized capsaicin (60 μmol/L, n = 15; Fig 1, *A*) and citric acid (300 mmol/L, n = 12-15; Fig 1, *B*).

### Effect of theophylline on C-fiber activation by capsaicin *in vivo*

**Fig 2:**
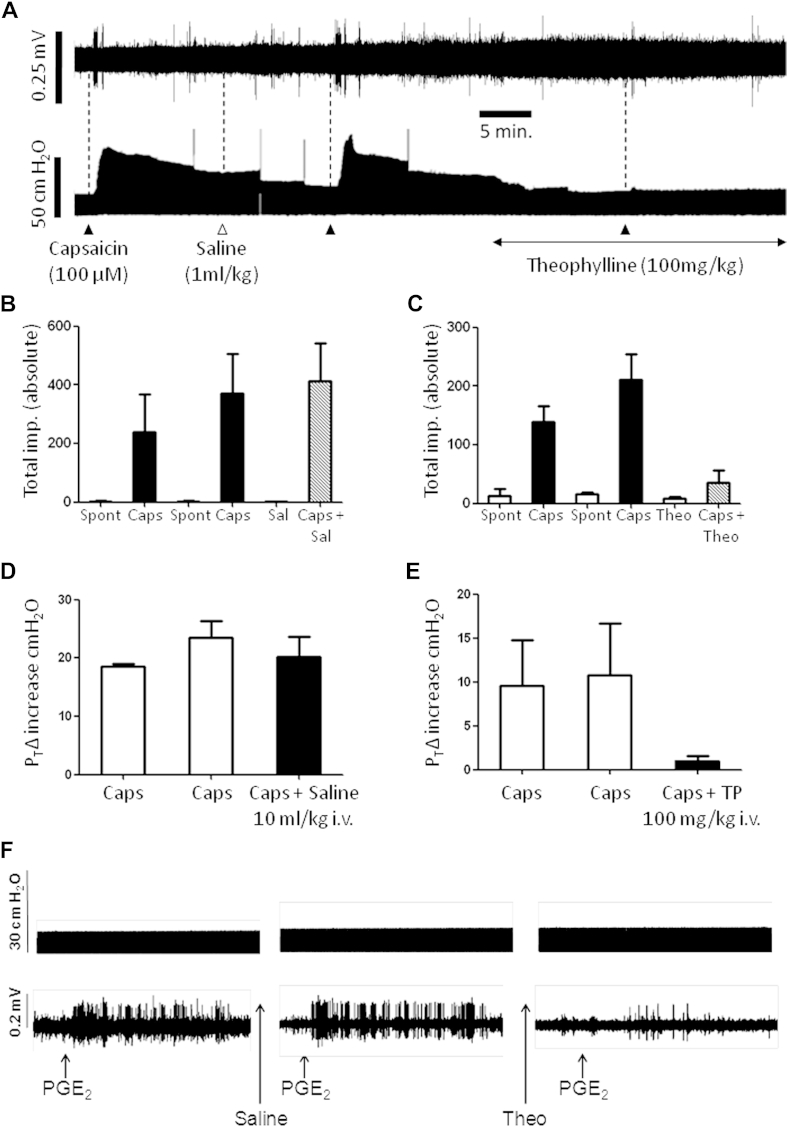
Effect of theophylline on airway vagal sensory afferent nerve firing and bronchospasm. **A,** Firing of a single vagal C-fiber *(top trace)* and simultaneous airway/tracheal pressure change *(bottom trace)*. Capsaicin (100 μmol/L aerosol for 15 seconds, *solid triangles*), saline (*Sal*; 10 mL/kg, *open triangles*), or theophylline (100 mg/kg administered intravenously) was administered as indicated. **B-E,** Histograms representing the average total impulse count over time (5-minute window from nebulization) recorded from single vagal C-fibers or average changes in airway/tracheal pressure in response to capsaicin in the presence of saline (Fig 2, *B* and *D*) or theophylline (Fig 2, *C* and *E*), respectively. Data are expressed as means ± SEs. *P* < .05 after analysis with the paired *t* test (n = 3). **F,** Example recording of the effect of theophylline on PGE_2_ (100 μg/mL, aerosol for 1 minutes)–induced changes in airway/tracheal pressure change *(top trace)* and simultaneous vagus nerve single C-fiber firing *(bottom trace)*.

Capsaicin, when nebulized into the lungs of anaesthetized guinea pigs, induced a burst of vagal C-fiber firing that was significantly reduced by theophylline (Fig 2, *A*-*C*). Theophylline administration *per se* did not modify the spontaneous firing observed in the vagal C-fibers (Fig 2, *C*). Capsaicin also induced a prolonged bronchoconstriction (which followed the C-fiber firing) that was almost entirely abolished in the presence of theophylline (Fig 2, *A*, *D*, and *E*). The possibility exists that some of the C-fiber firing could be induced indirectly after the bronchoconstrictor response elicited by capsaicin and that theophylline is merely acting as a bronchodilator. Aerosolized prostaglandin E_2_ (PGE_2_) was also used to induce bursts of C-fiber firing to address this concern. Action potential trains were obtained that were, on average, similar in total duration to capsaicin and were not associated with any bronchoconstriction (Fig 2, *F*, top panel). Theophylline significantly decreased PGE_2_-induced firing (Fig 2, *F*, bottom panel).

### Characterizing theophylline inhibition of vagal axon excitation

**Fig 3:**
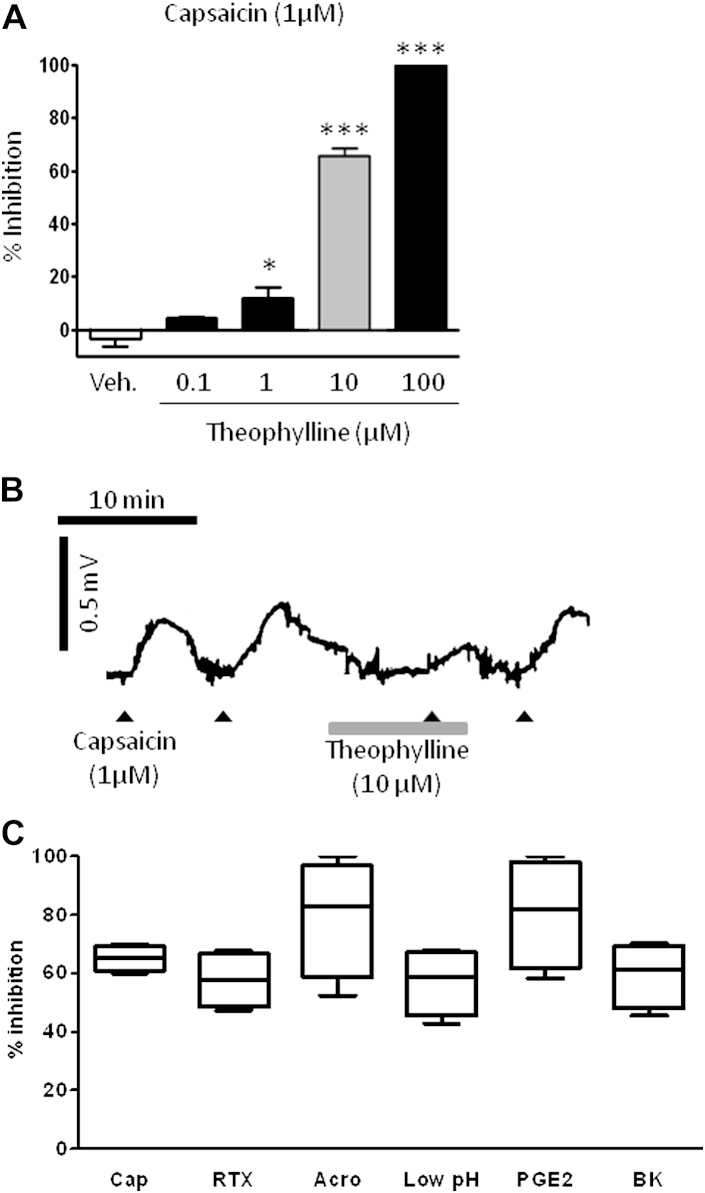
Effect of theophylline on vagus nerve depolarization. **A,** Effect of theophylline or vehicle (0.1% dimethyl sulfoxide, *white bar*) on capsaicin (1 μmol/L)–induced depolarization of the guinea pig vagus nerve (n = 6-13). The *gray bar* indicates the working concentration chosen for further experiments. *P < .05 and ***P < .001 using 1-way ANOVA with the Dunnett multiple comparison *post hoc* test. **B** and **C,** Representative trace (Fig 3, *B*) and box-and-whiskers diagram (Fig 3, *C*) showing the percentage inhibition of TRPA1 and TRPV1 agonist–induced vagal depolarization induced by theophylline (10 μmol/L, n = 4-6 per agonist) in which the *middle line of the box* indicates the median, the *lower line* indicates the minimum, and the *upper line* indicates the maximum. *Whiskers* indicate the 25th and 75th percentiles. Selected agonists were capsaicin (*Cap*; 1 μmol/L), acrolein (*Acro*; 300 μmol/L), resiniferatoxin (*RTX*; 3 nmol/L), acidity (pH 5, low pH), PGE_2_ (10 μmol/L), and bradykinin (*BK*; 3 μmol/L).

Theophylline inhibited capsaicin-induced depolarization of the guinea pig vagus axon in a concentration-dependent fashion (Fig 3, *A* and *B*). A submaximal concentration of theophylline (10 μmol/L) similarly inhibited depolarization to a range of sensory nerve stimulants, concentrations of which were selected from previous studies: capsaicin (1 μmol/L), PGE_2_ (10 μmol/L),^17,19^ acrolein (300 μmol/L),^16^ bradykinin (3 μmol/L),^21^ resiniferatoxin (3 nmol/),^16^ and low pH (pH 5)^16^ (Fig 3, *C*).

### Effect of potassium channel blockers on the inhibitory effect of theophylline on capsaicin-induced depolarization of guinea pig vagus

**Fig 4:**
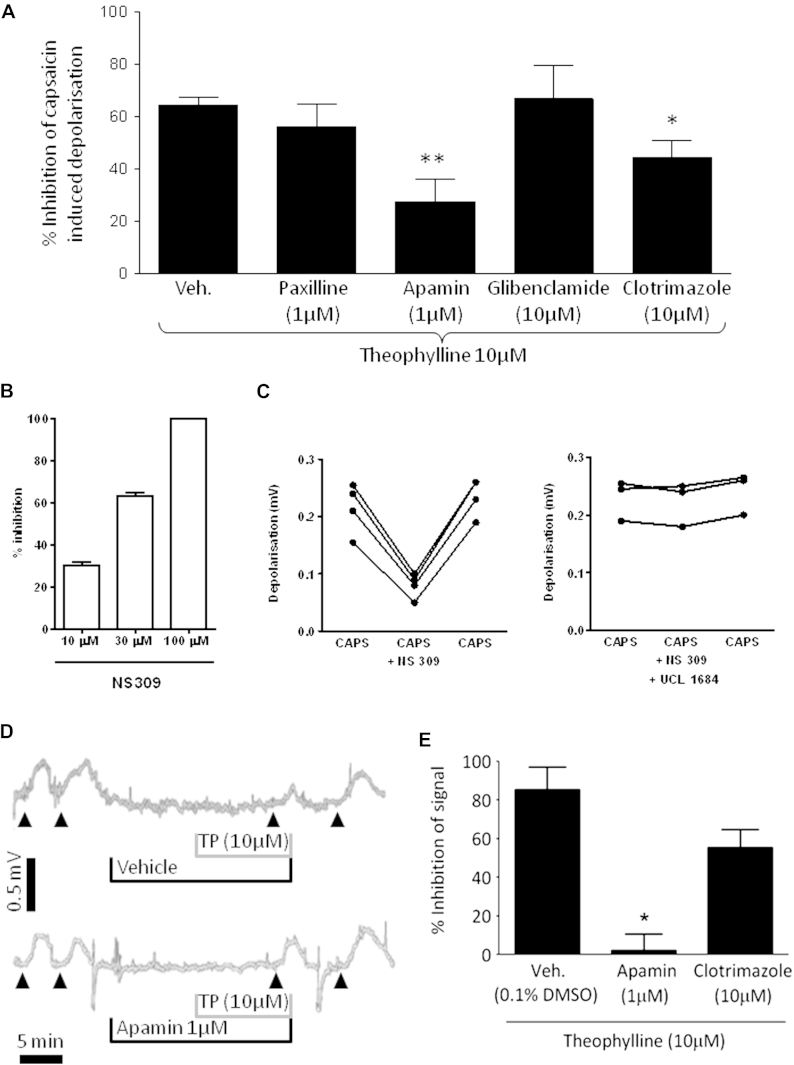
Inhibition of the effect of theophylline on guinea pig vagus sensory nerve depolarization. **A,** Theophylline inhibition of capsaicin (1 μmol/L)–induced depolarization in the presence of various ion channel blockers: paxalline (BK_Ca_ channel, n = 5), apamin (SK channel, n = 5), clotrimazole (IK channel, n = 4), glibenclamide (K_ATP_ channel, n = 5), and vehicle (0.1% dimethyl sulfoxide, n = 7). Data are expressed as means ± SEs. *P < .05 and **P < .01 using 1-way ANOVA with the Bonferroni multiple comparison *post hoc* test. **B,** Inhibitory effect of NS309 (SK channel opener) on capsaicin-induced depolarization (n = 4-6). **C,** Point and line graphs showing the inhibition of capsaicin-induced depolarization by NS309 (30 μmol/L; *left panel*, n = 4) and the reversal induced by preincubation with the SK blocker UCL1684 (10 μmol/L; *right panel*, n = 3). **D,** Representative traces showing responses to capsaicin (1 μmol/L, *solid triangles*) recorded by using human vagus nerve before and after incubation with theophylline (10 μmol/L) in the presence of vehicle (0.1% dimethyl sulfoxide, *top trace*) or the SK channel blocker apamin (1 μmol/L, *bottom trace*, n = 4). **E,** Effect of vehicle, apamin, or clotrimazole on theophylline-induced inhibition of capsaicin-induced depolarization of human vagus nerve (n = 4). Data are expressed as means ± SEs. *P < .05 after analysis with the paired *t* test.

The general inhibitory effect of theophylline suggested that, rather than blocking a specific receptor, it could activate a mechanism that combats the depolarization of the neuronal membrane by decreasing its excitability. Using pharmacological agents at previously determined concentrations,^23^ we investigated the role of potassium channels on the inhibitory effect of theophylline. Preincubation with paxilline (a large-conductance calcium-activated potassium channel [BK_Ca_ channel] blocker) or glibenclamide (an ATP-sensitive potassium channel [K_ATP_ channel] blocker) did not modify the inhibitory effect of theophylline on capsaicin (1 μmol/L)–induced depolarization of the vagus. In contrast, apamin (a highly selective SK1, SK2, and SK3 channel blocker) and clotrimazole (a selective IK blocker) significantly decreased the inhibitory effect of theophylline (Fig 4, *A*). We reproduced the inhibitory effect observed with theophylline using NS309 (an SK channel opener), and this inhibition was significantly reduced in the presence of UCL1684 (10 μmol/L; an SK channel blocker; Fig 4, *C*).

### Effect of theophylline on human vagal sensory nerve activation

Theophylline significantly inhibited capsaicin-induced depolarization, an effect that was blocked in the presence of apamin but not clotrimazole (in contrast to the data generated in guinea pig vagus; Fig 4, *D* and *E*).

### Effect of theophylline on capsaicin-induced changes in [Ca^2+^]_i_ levels and membrane voltage in primary guinea pig sensory jugular neurons

**Fig 5:**
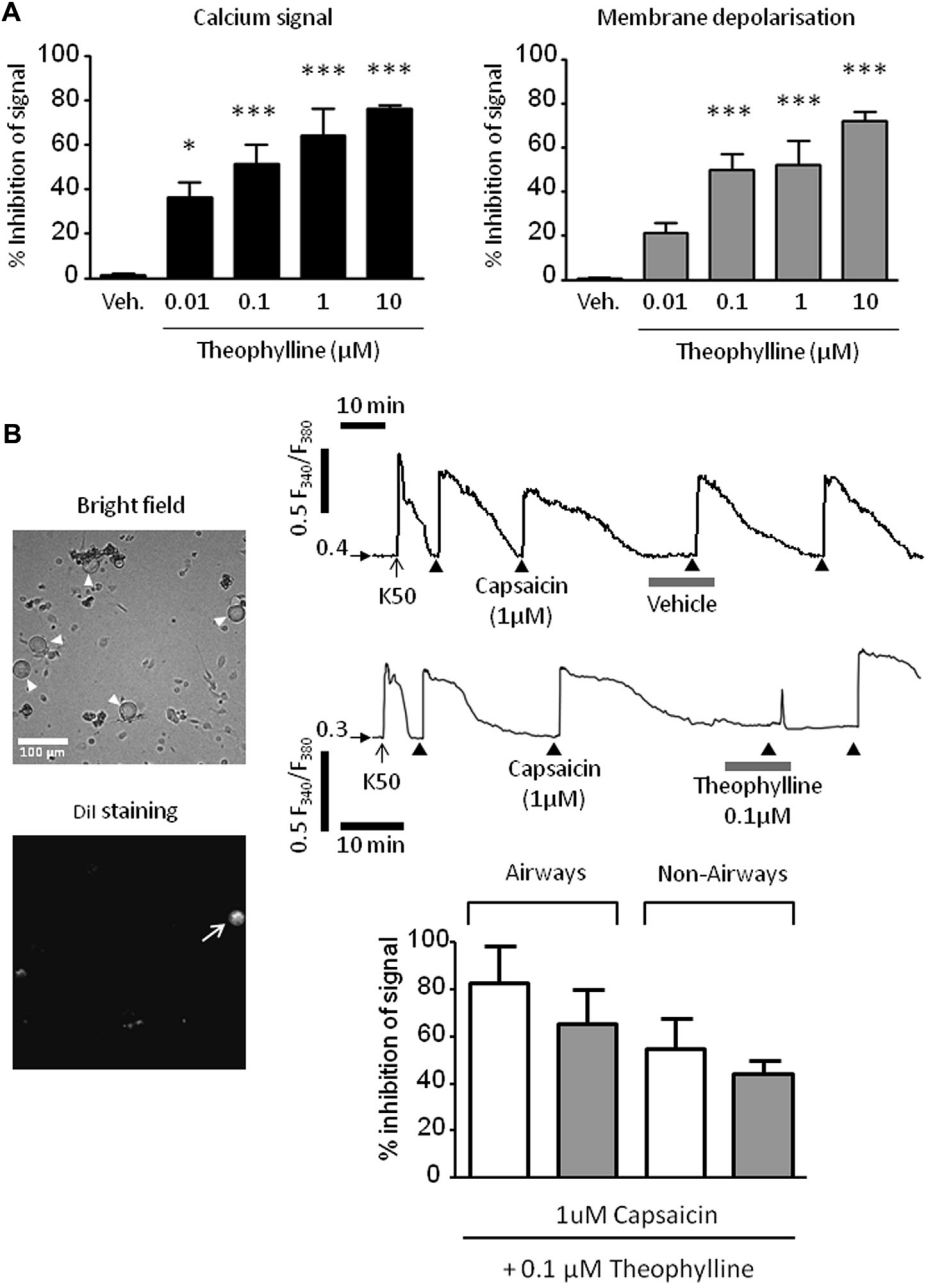
Inhibition of capsaicin-induced activation of vagal sensory ganglia. **A,** Concentration-related inhibitory effect of theophylline on capsaicin (1 μmol/L)–induced [Ca^2+^]_i_ signal *(left panel)* and membrane depolarization *(right panel)* of jugular sensory neurons (n = 10-22 per concentration; N = 4 animals). Data are presented as means ± SEs. *P < .05 and ***P < .001 using 1-way ANOVA with the Bonferroni multiple comparison *post hoc* test. **B,***Left panel*, The *top image* shows primary cultured neurons dissociated from jugular sensory neurons in bright field *(open triangles)*. Of these 5 neurons, only 1 neuron stained with retrograde tracer DiI originated from the airway (*bottom image*, indicated by *white arrow*). *Top right panel*, Trace (F_340_/F_380_ ratio, vertical left scale) showing [Ca^2+^]_i_ levels recorded with Fura2 for the airway neuron exposed to potassium solution (*K50*; KCl 50 mmol/L) and then capsaicin *(solid triangles)* before and after incubation with theophylline. *Lower right panel*, Histogram showing inhibition of capsaicin-induced [Ca^2+^]_i_ signal and membrane depolarization by theophylline in sensory neurons (N = 4 animals; n = 6 airway and n = 15 nonairway). Data are presented as means ± SEs.

The free resting calcium level was calculated under our experimental conditions to be between 60 and 132 nmol/L (average, 122 ± 28 nmol/L; n = 5) at rest in primary cultured jugular neurons. This is lower than the estimated median effective concentration of calmodulin (an SK channel calcium sensor) for calcium (approximately 300-500 nmol/L) and suggests that the SK channel will not be activated under basal conditions. Capsaicin (1 μmol/L) in extracellular solution (ECS) was applied through a fast perfusion for 30 seconds, and the response was recorded until the signal returned to baseline. Theophylline produced a concentration-dependent inhibition of capsaicin-induced [Ca^2+^]_i_ increase and membrane depolarization (Fig 5, *A*). From these experiments, a working concentration of theophylline (0.1 μmol/L) was chosen as an approximate median effective concentration (an effective concentration that blocks 50% of the maximal response) for future studies. Theophylline also inhibited capsaicin-induced excitation of airway sensory neurons originating from the jugular ganglion (Fig 5, *B*). The inhibition achieved was similar to the inhibition obtained on the response of all neurons (both airway-specific and nonairway neurons).

### Effect of theophylline on SK and IK channel potassium currents in the jugular ganglia and analysis of the direct effect of theophylline on calcium-activated potassium channels

**Fig 6:**
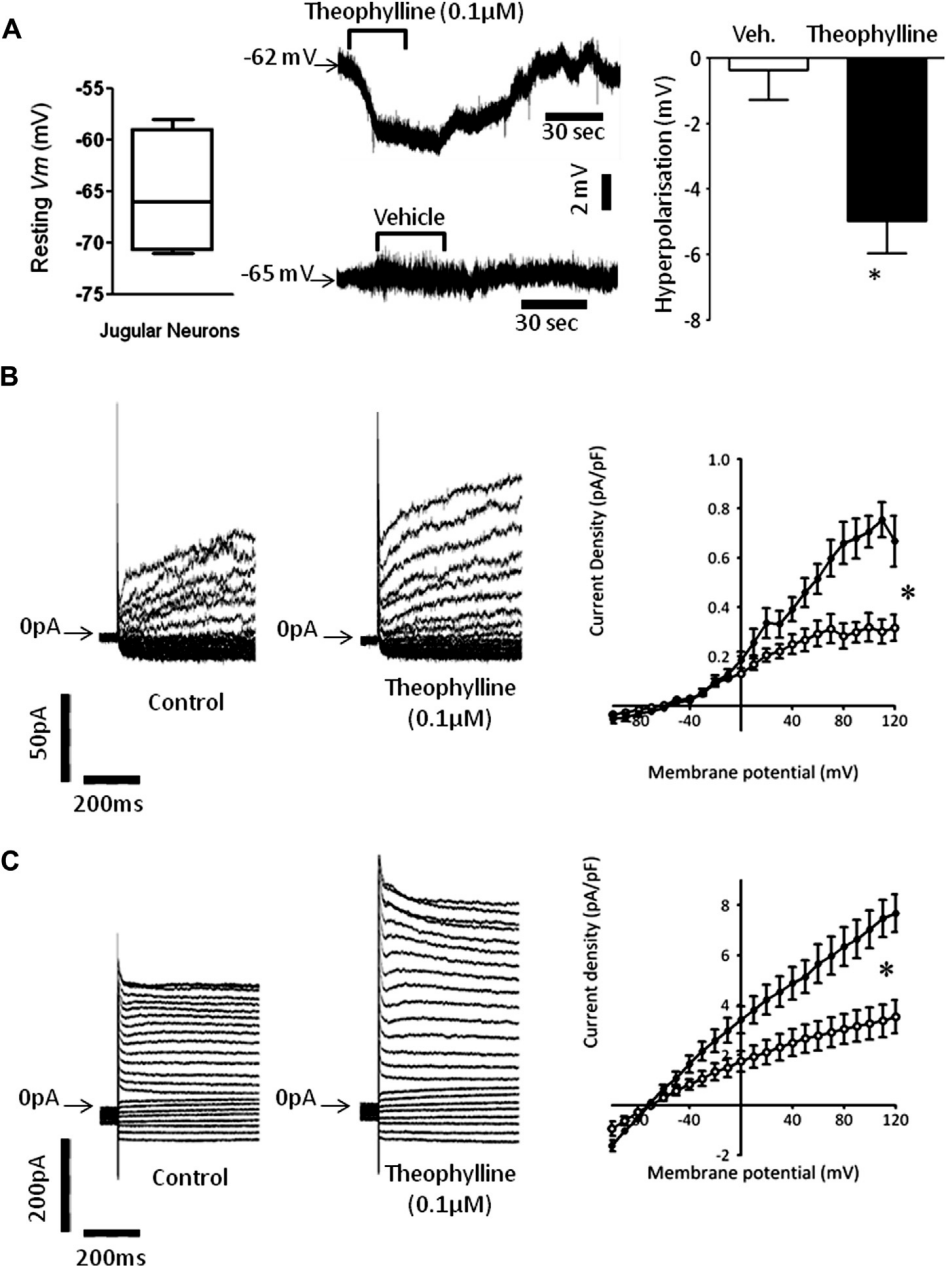
Theophylline hyperpolarizes neurons by potentiating calcium-activated potassium currents. **A,***Left panel*, Box-and-whisker graph showing the range of membrane potentials at rest. *Middle panel*, Trace of membrane potential change recorded during incubation with vehicle or theophylline in current clamp mode (*I* = 0) and perforated patch whole-cell configuration. *Right panel*, histogram of the averaged resting membrane potential change recorded on 4 cells. Data are expressed as means ± SEs. *P < .05 compared with the control (vehicle, 0.1% dimethyl sulfoxide) using the Mann-Whitney *U* test. **B,** Currents were elicited by 800-ms square depolarizing pulses from −100 mV up to +120 mV with a 10-mV increment and −70 mV holding potential. The zero current levels are indicated by an *arrow* on the left of the traces. *Left panel*, Clotrimazole (10 μmol/L)–sensitive current obtained under control (0.1% dimethyl sulfoxide) conditions and after incubation with theophylline. *Right panel*, Current density-voltage relationships in the control condition (*open circles*, n = 4) and after incubation with theophylline (0.1 μmol/L, *solid circles*, n = 4). *P < .05. **C,** *Left panel*, Apamin (1 μmol/L) sensitive current obtained control condition (0.1% dimethyl sulfoxide) and after incubation with theophylline. *Right panel*, Current density-voltage relationships under control conditions (*open circles*, n = 4) and after incubation with theophylline (0.1 μmol/L, *solid circles*, n = 4). Data are expressed as means ± SEs. Current density amplitudes were compared by using 2-way ANOVA and Bonferroni *post hoc* tests. *P < .05.

The effect of theophylline on the resting membrane potential of primary jugular neurons was investigated with a patch clamp. A perforated patch configuration was used to preserve the intracellular content and [Ca^2+^]_i_ levels, and the cells were constantly perfused with ECS at 37°C. Theophylline significantly hyperpolarized the membrane (Fig 6, *A*). Because the SK and IK channel blockers apamin and clotrimazole blocked the inhibitory effect of theophylline on vagus nerve activation, we investigated the effect of theophylline on these currents. However, before recording the potassium currents, the cell perfusion was switched to calcium-free modified ECS to prevent calcium entry into the cell during depolarization and to maintain the [Ca^2+^]_i_ concentration at its resting value. Modified ECS was switched back to ECS (calcium, 2.5 mmol/L) for all washout periods to prevent the cells from depleting their calcium stores. Sodium and calcium currents were blocked with nifedipine, tetrodotoxin, and salt substitution with choline. We used recording subtraction to isolate the potassium current generated by the SK and IK populations of channels: recordings made in the presence of clotrimazole (10 μmol/L, a selective IK channel blocker) or apamin (1 μmol/L, a selective SK1, SK2, and SK3 blocker) in modified ECS were subtracted from their respective control recordings also done in modified ECS to obtain the clotrimazole- or apamin-sensitive currents (ie, the portion of the whole potassium current blocked by each specific blocker). Fig 6, *B* (left panel), shows clotrimazole-sensitive currents obtained in control conditions (modified ECS + vehicle − theophylline free) and after incubation with theophylline (0.1 μmol/L) in modified ECS. The current density-voltage relationship displayed in the right panel indicates that the clotrimazole-sensitive current (IK) was significantly increased after incubation with theophylline (0.1 μmol/L) at resting calcium levels (n = 4). However, the difference between the 2 curves only reached significance (*P* < .05) when the potential reached +60 mV with a 56% current density increase from 0.25 ± 0.04 pA/pF up to 0.39 ± 0.05 pA/pF at +60 mV (n = 4). The apamin-sensitive current (Fig 6, *C*) was also significantly increased after incubation with theophylline (0.1 μmol/L) at resting calcium levels (n = 4). The difference between the 2 current-voltage curves reached significance for a membrane potential of −30 mV (*P* < .05), with a 105% current density increase from 1.06 ± 0.10 pA/pF up to 2.17 ± 0.21 pA/pF at −30 mV (n = 4).

**Fig 7:**
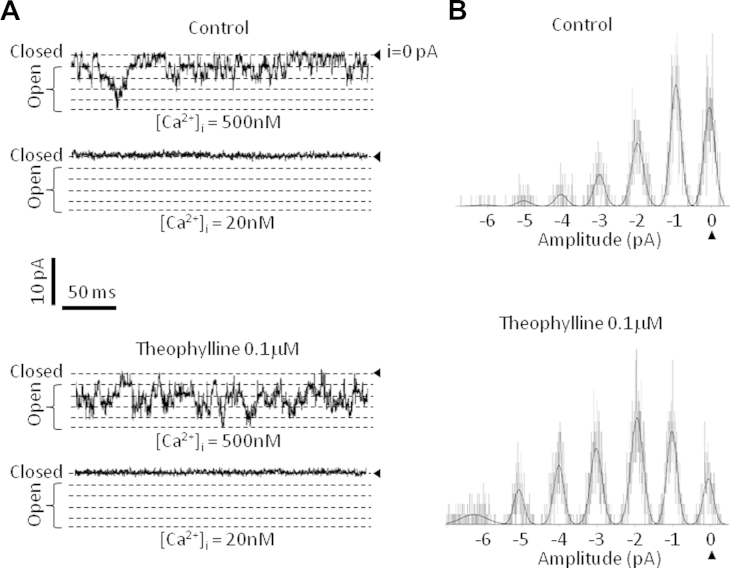
Theophylline directly activates the SK channel independently from intracellular machinery. Application of theophylline increases the open probability of SK channels in the presence of calcium. **A,** Traces acquired at −100 mV of channel openings recorded from the same patch of membrane in the presence of the control (0.1% dimethyl sulfoxide, *top panel*) and in the presence of theophylline *(bottom panel)* with high or low free calcium levels ([Ca^2+^]_i_). The channel closed state (i = 0 pA) is indicated on the right by a *solid triangle*. Open states are indicated with a *bracket on the left* and identified by a *dotted line*. Current and time scales are indicated between both panels by *black bars*. **B,** Normalized amplitude histograms of the single-channel traces from the patch displayed in Fig 7, *A*, recorded at high calcium levels over 30 seconds and fitted by using a Gaussian function. The current level corresponding to the closed state of the channel (0 pA) is indicated by a *solid triangle*. The amplitude scale is indicated below the histograms on the *x-axis*.

In single-channel inside-out experiments an excised patch of membrane was successively exposed to control solutions containing vehicle and supplemented with high and low free calcium concentrations. The patch was then exposed to theophylline (0.1 μmol/L) in solutions containing high and low calcium concentrations to compare the open probability of the same patch in all 4 conditions. Fig 7, *A*, shows the opening of the channels from a patch of membrane with or without theophylline in the bath. The theophylline effect appeared to be calcium dependent because it did not induce any further channel opening in low calcium. Unitary current was not changed after theophylline application (0.97 ± 0.16 pA in control vs 1.01 ± 0.17 pA in the presence of theophylline, n = 5), whereas the channel open probability increased by 62% ± 7% at −100 mV voltage in the presence of 500 nmol/L free calcium (Fig 7, *B*).

### Effect of theophylline on enhanced cough caused by capsaicin

**Fig 8:**
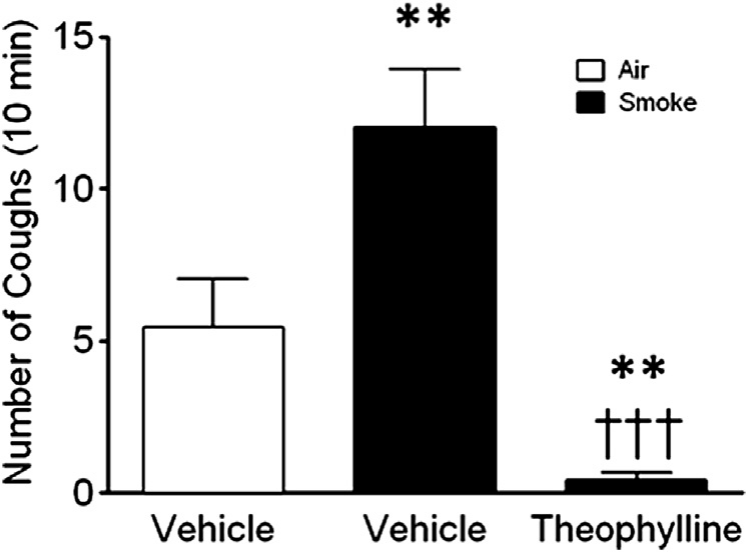
Theophylline inhibits cigarette smoke–enhanced capsaicin-evoked cough. Histograms showing cough counts per 10 minutes in response to nebulized capsaicin (30 μmol/L for 5 minutes) for air-exposed *(white bar)* or smoke-exposed *(black bars)* animals. Data are expressed as means ± SEMs (n = 12). **P < .01 when comparing the response of air-exposed and smoke-exposed animals to capsaicin. †††*P* < .001 when comparing smoke-exposed animals' response to capsaicin after treatment with theophylline (100 mg/kg administered intraperitoneally 1 hour before the experiment) or vehicle alone (0.5% methylcellulose and 0.2% Tween 80 in saline).

In this model exposure to cigarette smoke significantly enhanced cough induced by capsaicin (Fig 8). Pretreatment with theophylline almost abolished this enhanced cough.

## Discussion

Theophylline has been used in the treatment of asthma and COPD for many years and is still prescribed for its bronchodilator activity and low cost.^1^ However, the need for therapeutic drug monitoring and the hesitancy of clinicians to use theophylline because of a perceived poor side effect profile has resulted in the drug not being widely used. In the developed world it is more commonly used as a third-line treatment, particularly in patients with poorly controlled disease.^1^ Despite current COPD guidelines indicating that theophylline is of limited value in the routine management of COPD, many controlled clinical trials support its utility in patients with stable COPD^24,25^ and also observed worsening of the clinical state when theophylline was withdrawn. Furthermore, it has also been recommended for the treatment of cough in patients with COPD.^8,26^ In children and adults with poorly controlled asthma, theophylline significantly improved symptom scores for cough and wheeze compared with placebo,^7^ and it has been shown to be effective for treating angiotensin-converting enzyme inhibitor–related cough.^9^

The antitussive activity of another methylxanthine, theobromine, has also been demonstrated in preclinical and clinical studies.^5,6^ However, in previous studies it was suggested that this property was particular to theobromine and not related to its methylxanthine activity.^27,28^ However, the data presented suggest that theophylline might present a readily available and inexpensive alternative therapy to opioids for the treatment of cough, which might be better tolerated and could be prescribed for chronic cough throughout the developing and developed world. The aim of these studies was to test the hypothesis that theophylline possesses general antitussive activity by inhibiting sensory nerve activity, thus highlighting a new application for an old drug and potentially identifying a novel antitussive mechanism that can be targeted by new therapeutic agents with a more optimal therapeutic ratio.

In the present study the antitussive activity of theophylline was demonstrated in a conscious guinea pig cough model against both capsaicin and citric acid challenge, which are commonly used tussive stimuli in human challenge studies.^29^ This is consistent with a previous study that found significant inhibition of citric acid–induced cough using theophylline at 10 mg/kg.^5^ To confirm an inhibitory effect on airway vagal afferents, we demonstrated an effect of theophylline on capsaicin-induced excitation of airway sensory neurons originating from the jugular ganglion. Furthermore, single-fiber recording studies demonstrated an inhibitory action of theophylline on capsaicin-induced action potential generation in C-fiber afferents. The inhibitory activity on capsaicin-sensitive C-fibers was also accompanied by an inhibition of capsaicin-induced bronchospasm, confirming the bronchodilator activity of theophylline at a similar dose range. Antitussive activity of compounds, such as β_2_-adrenoceptor agonists,^23^ has been attributed previously to bronchodilator activity.^30^ Aerosolized PGE_2_ was also used to induce bursts of C-fiber firing to validate our hypothesis that theophylline has a direct inhibitory effect on C-fiber afferents *in vivo*, rather than acting simply as a bronchodilator. Theophylline inhibited the action potential trains obtained to aerosolized PGE_2_, an agent that activates C-fibers without evoking bronchoconstriction.

In further experiments to circumvent the potentially confounding bronchodilator properties of theophylline and to explore its pharmacology in more detail, an isolated vagus nerve preparation was used to study its effects directly on sensory nerve depolarization, which is a measure of sensory nerve activity.^20,31^ This technique has previously been characterized, and the vagus axon has been shown to respond to a range of agents known to cause cough both in preclinical models and human cough studies. Furthermore, it provides the opportunity to conduct a comprehensive pharmacologic assessment of the direct action of drugs on the vagus without the pharmacokinetic and numerous other considerations that limit the interpretation of *in vivo* data. Although there are certain caveats to using this technique, which have been extensively discussed in other publications,^19^ it appears that responses in guinea pig and human tissues are comparable and that the results translate to *in vivo* models, suggesting that this technique is amenable for use in studying the mechanisms involved in sensory nerve modulation.^16,17,21^ Theophylline inhibited capsaicin-induced depolarization of both guinea pig and human isolated vagus nerve preparations. These data and the *in vivo* single-fiber recording studies would suggest that the antitussive effect of theophylline is due to a peripheral action on airway vagal sensory nerve activity and not its bronchodilator activity.

Even in its recognized clinical role as a bronchodilator, the underlying mechanism of action of theophylline is unclear. However, it is known that theophylline is a weak and nonselective phosphodiesterase (PDE) inhibitor that breaks down cyclic nucleotides in the cell, leading to an increase in cyclic AMP and cyclic GMP concentrations. Furthermore, there have been some reports suggesting that certain PDE4 inhibitors can inhibit cough to inhaled capsaicin in normal guinea pigs^32,33^ and in allergic guinea pig models of asthma.^32,34-36^ However, in contrast, a lack of effect of PDE4 inhibition on citric acid–induced cough in normal guinea pigs has also been reported.^35^ It is unlikely that PDE inhibition can account for the inhibitory effect of theophylline on sensory nerve depolarization and thereby its antitussive activity, given that theophylline is reported to inhibit PDE1, PDE2, PDE3, PDE4, and PDE5 with inhibitory concentration of 50% (IC_50_'s) values of 280, 55, 98, 100 and 630 μmol/L in human cell–based assay systems,^37-39^ whereas theophylline inhibited vagal sensory nerve depolarization in this study with an approximate IC_50_ of 3 μmol/L. However, an *in vivo* inhibitory effect of theophylline on PDE4 activity cannot be completely ruled out with regard to its *in vivo* antitussive activity. At therapeutic concentrations, theophylline can also act as an antagonist of adenosine A_1_, A_2_, and A_3_ receptors. Furthermore, adenosine has been shown to activate sensory afferents through A_1_ and A_2a_ receptors.^40^ However, we have established that theophylline inhibits depolarization to a variety of tussive agents, including transient receptor potential (TRP) V1 agonists (capsaicin and resiniferatoxin), TRPA1 ligands (acrolein), PGE_2_, bradykinin, and low pH, suggesting that theophylline acts as a general inhibitor of neuronal excitability rather than an adenosine receptor antagonist.

This study shows that theophylline decreases the excitability of sensory nerves and thereby induced cough. Further investigation of the mechanism of action using the isolated vagus nerve preparation and a pharmacological approach demonstrated it to be an activator of calcium-activated potassium channels, which are involved in the control of many neuronal processes, including regulation of the firing rate along axons.^41^ We showed the effect of theophylline was due to activation of apamin-sensitive SK channels (SK1, SK2, and SK3) and, to a lesser extent, IK channels in guinea pig and human vagal afferents. These data are consistent with articles suggesting that these channels are expressed in human peripheral sensory nerves.^42^ Inhibition by theophylline of the calcium and voltage signals to the tussive agent was not preceded by an increase in [Ca^2+^]_i_ levels, indicating that the concentrations of theophylline used did not modify basal [Ca^2+^]_i_ levels in isolated sensory neurons.

To investigate this in more detail, we used a perforated patch clamp system to preserve all intracellular content while preventing calcium influx, which would change basal [Ca^2+^]_i_ levels through calcium-induced calcium release. Our electrophysiological data indicated that theophylline activates guinea pig IK and apamin-sensitive SK channels at resting [Ca^2+^]_i_ levels to inhibit the depolarization of jugular neurons and are consistent with data from Schroder et al^43^ demonstrating that methylxanthines activate potassium channels without an increase in [Ca^2+^]_i_ levels. Although the activation was not secondary to an increase in [Ca^2+^]_i_ levels, single-channel recording suggested a requirement for calcium to be present. The reduced activation of the IK channel in comparison with the SK channel and the absence of an effect on the BK_Ca_ channel could be linked to the different calcium sensitivity and voltage dependence of these channels. Indeed, IK channel open probability is poorly dependent on voltage and is largely regulated by [Ca^2+^]_i_. Its opening concentration threshold can vary over a range of concentrations depending on the conditions but remains higher than for SK. The BK_Ca_ channel open probability is voltage dependent and requires high [Ca^2+^]_i_ levels, suggesting that theophylline's effect at the concentration used was not enough to enhance activation of the BK_Ca_ channel by [Ca^2+^]_i_ levels.^44,45^ On the other hand, the SK channel is highly sensitive to calcium and voltage insensitive. This gives it the ability to open at low [Ca^2+^]_i_ levels independent from the membrane potential, and it has been shown to be important in controlling neuronal excitability and firing rate along axons.^46^ The wide range of calcium sensitivity for IK channel gating and the need for high depolarization/increase in [Ca^2+^]_i_ levels to open BK_Ca_ suggests that the resting [Ca^2+^]_i_ levels under these conditions would not be sufficient for theophylline to open these channels. Theophylline binding to the SK channel could either directly modify the open probability independently from the gating or increase the binding of calcium, thereby acting as calcium sensitizer. However, the possible activation of the IK channel, BK channel, or both by theophylline, when sensory neurons are activated, [Ca^2+^]_i_ levels are increased, and the membrane is depolarized, also remains a possibility. As mentioned previously, an *in vivo* inhibitory effect of theophylline on PDE4 activity cannot be completely ruled out with regard to its *in vivo* antitussive activity, and in fact, protein kinase A activation has been shown to indirectly activate the SK channel through an increase in [Ca^2+^]_i_ levels.^47^ However, although protein kinase A regulates IK channels in oocytes,^48^ this is not apparent in mammalian cells.^43,49^ Therefore the possibility exists that the effect of theophylline on SK channels could be mediated indirectly through an inhibition of PDE activity.

Recently, ion channels of the TRP class, such as TRPV1, have been implicated in the heightened cough sensitivity seen in disease, and several studies have demonstrated an increase in cough reflex sensitivity to capsaicin challenge in patients with COPD.^50,51^ These data might indicate that TRPV1 channels could be key effectors of tussive responses in this disease and that these channels could be associated with long-term potentiation of the cough reflex. Capsaicin-induced tussive responses are also increased in our conscious guinea pig model after cigarette smoke exposure, modeling the human pathophysiology. The ability of theophylline to decrease this exaggerated tussive response suggests that this therapeutic approach might be effective in the treatment of cough associated with COPD.

In conclusion, the antitussive activity of theophylline under normal and disease conditions highlights a previously unrecognized beneficial property of theophylline, and the discovery of its mechanism of action might lead to the development of optimized compounds with a reduced side effect potential.

**Key messages**

• Clinical studies have suggested that theophylline acts as an antitussive agent in a range of conditions; however, the mechanism of action is not known.
• Here we show that theophylline inhibits sensory nerve activation (human and guinea pig) to a range of tussive stimuli through increasing the open probability of calcium-activated potassium channels (predominantly the SK channel).
• These findings could highlight a previously unrecognized beneficial property of theophylline and lead to the development of optimized antitussive compounds.

## Supplementary data

Online Methods and Online References E1-E7
