# Supplementary material for: Theophylline inhibits the cough reflex through a novel mechanism of action
Source: J Allergy Clin Immunol. 2014 Jun;133(6):1588–98. doi: 10.1016/j.jaci.2013.11.017 (PMC4048545; doi:10.1016/j.jaci.2013.11.017)
Supplement: Online Methods and Online References E1-E7 [file mmc1.docx]

**Online Data Supplement**

**Theophylline inhibits sensory nerve activation and the cough reflex via activation of small and intermediate calcium-activated potassium channels.**

Eric Dubuis, Michael A. Wortley, Megan Grace, Sarah A. Maher, John J. Adcock, Mark A. Birrell, Maria G. Belvisi,

**METHODS**

*Effect of theophylline on citric acid and capsaicin evoked cough in conscious guinea pigs*

Conscious, unrestrained guinea pigs were placed in individual plastic transparent whole-body plethysmograph chambers (Buxco, Wilmington, NC, USA), and cough was detected as previously described (E1, E2). Guinea pigs were dosed with vehicle (0.5% methylcellulose + 0.2% Tween 80 in 0.9% sterile saline) or theophylline (Sigma Aldrich, 0.3, 3, 30, or 100 mg/kg, 10 ml/kg) intraperitoneally 1h prior to exposure experiments. Cough was evoked by exposing the animals to aerosolised citric acid (0.3M) for 10 min or capsaicin (60mM) for 5 min. Coughs were detected both by pressure change and sound recorded by the analyser and confirmed by a trained observer and counted for a 10 min period from the start of the drug delivery (E1, E2).

*Effect of theophylline on capsaicin induced firing of single fibre afferents and bronchospasm in vivo*

Guinea-pigs were anaesthetized with urethane (1.5 g/kg) intraperitoneally. If required, anaesthesia was supplemented with additional urethane. The trachea was cannulated and pressure was measured with an air pressure transducer (SenSym 647) connected to a side arm of the tracheal canula. Blood gases and pH were maintained at physiological levels by artificial ventilation (Ugo Basile small animal ventilator), with a tidal volume of 10 ml/kg and 50-60 breaths per min of laboratory air. The right jugular vein and carotid artery were cannulated for injecting drugs and measuring systemic arterial blood pressure (Gould P23XL transducer), respectively. Body temperature was continuously monitored with a rectal thermometer and maintained at 37ºC with a heated blanket and control unit (Harvard Apparatus). Animals were paralysed with vecuronium bromide, initially administered at a dose of 0.10 mg.kg^-1^ i.v., followed every 20 min with 0.05 mg.kg^-1^ i.v. to maintain paralysis. The depth of anaesthesia was frequently assessed by monitoring the response of heart rate and blood pressure to noxious stimuli. Both cervical vagus nerves were located, via a cervical incision, and dissected free from the carotid artery, sympathetic and aortic nerves; both vagus nerves were cut at the central end. The left vagus nerve was used for sensory nerve fibre recording and was cleared of its surrounding fascia. The skin and muscle in the neck at either side of the incision were lifted and tied to a metal ring to form a well, which was filled with light mineral oil. Bipolar Teflon-coated platinum electrodes (exposed at the tips) were used for recording purposes, using fascia positioned on one electrode for a reference. The vagus nerve was placed on a small black perspex plate to facilitate subsequent dissection. Thin filaments of nerve were teased from the vagus nerve, under a binocular microscope, until a single active unit or one of not more than two or three units was obtained, and placed on the second electrode. Action potentials were recorded in a conventional manner using electrodes connected to a pre-amp headstage (Digitimer NL100K). The signal was amplified (x5000, Digitimer NL104), filtered (LF30Hz – HF8.5kHz, Digitimer NL125) and passed through a Humbug noise reducer (AutoMate Scientific) before input sampling and recording. All signals were sampled (50 kHz) and recorded using the Spike 2 software data acquisition system via a CED Micro1401 interface. The software allowed pulse train counting over selected time periods. In addition, monitoring of the input signal to the Spike software was also carried out on a digital storage oscilloscope (Tektronix DPO 2012). The input signal was also fed through an audio amplifier to a loud speaker. All animals were killed at the end of the experiments with an overdose of pentobarbitone.

Conduction velocities were measured to distinguish slow conducting non-myelinated C-fibres from fast conducting myelinated A-fibres by stimulating the vagus nerve close to the thorax with bipolar silver electrodes, using a supra threshold voltage at 0.5 ms, 1 Hz (Grass stimulator). The corresponding action potential was recorded in the nerve fibre under observation. The stimulus and the recorded action potential were captured on the Spike software in a single sweep and the time interval between them was measured to calculate the velocity using the distance from the cathode stimulating electrode and the recording electrode. Aerosols were generated by an Aerogen nebulizer (Buxco Nebulizer Control – 5) connected to the ventilator and arranged so that the inspired air passed through the medication chamber before entering the lungs of anaesthetized animals via the tracheal cannula. Single vagal nerve fibres were identified as originating from the three groups of airway sensory nerve endings, i.e., slowly adapting stretch receptors (SARs), irritant receptors (rapidly adapting stretch receptors, RARs, Aδ-fibres) and pulmonary/bronchial C-fibre receptors using several criteria (E3). These included pattern of spontaneous discharge, response to hyperinflation and deflation, adaptation indices (AIs), and response to capsaicin aerosol administration and conduction velocities. As a rule, a receptor that had no obvious pattern to the spontaneous activity (often very sparse), didn’t respond to hyperinflation or hyperdeflation, and responded to capsaicin aerosol was considered for further investigation. Finally, verification of a C-fibre was confirmed at the end of the experiment by determining conduction velocity. After surgery the animals were allowed to stabilize for at least 30 min. Following identification of a lung afferent fibre and its sensitivity to capsaicin aerosol, the ensuing protocol was pursued: After a control baseline recording of at least 2 min, capsaicin (100 µM) was administered by aerosol for 15 s and the changes in fibre activity, intra-tracheal pressure and blood pressure were continuously recorded until baseline or a steady state was re-established. After an interval of 10 min, saline (vehicle, 10 ml/kg) was injected i.v. while recording variables. After another interval of 10 min, the capsaicin aerosol was repeated. Ten min later, either saline (10 ml/kg) or theophylline (100 mg/kg i.v.) were injected i.v. followed 10 min later by the capsaicin aerosol.

*Effect of theophylline on depolarisation of the vagus nerve preparation to various tussive agents.*

Guinea pigs were culled with an overdose of pentobarbitone (200 mg/kg i.p.). The two vagus trunks were carefully dissected free and placed in Krebs–Henseleit solution (Krebs) solution (in mM: NaCl 118; KCl 5.9; MgSO_4_ 1.2; NaH_2_PO_4_ 1.2; CaCl_2_ 2.5; glucose 6.6; NaHCO_3_ 25.5,) bubbled with 95% O_2_ / 5% CO_2_. The vagus was carefully cleared of connective tissue and desheathed under a dissecting microscope, with care taken to avoid stretching or damaging it, and then cut into 15-20 mm long segments. Throughout the dissection, the nerve remained in oxygenated cold Krebs. The segments of vagal nerve were mounted in a ‘grease-gap' dual recording chamber system as previously described (E4, E5). Briefly, the nerve segment was inserted longitudinally through a narrow cylindrical chamber (2 mm diameter, 10 mm length) in a Perspex chamber with one end being the test chamber and the second end being the reference chamber. Both chambers were electrically and chemically isolated by injection of high electrical resistant inert petroleum jelly between them through a side arm once the nerve was in place. One end of the nerve segment was apparent in the test chamber which was constantly perfused with oxygenated 37°C Krebs at a flow rate of 2 ml/min. During experiments, perfusate was maintained at 37°C by the mean of in house designed water jacketed heat exchanger and change of solution was made through a manifold with minimum solution mixing and a delay of approximately 10 s. Drugs were applied at chosen concentrations into the test chamber only. The other nerve ending remained in the referenced chamber filled with oxygenated Krebs. Krebs filled borosilicate glass electrodes mounted on an Ag/AgCl pellet (World Precision Instruments) were put in contact at both ends of the nerve trunk and changes in surface charges between the test and reference chamber nerve ending were recorded using an extracellular potential differential amplifier (DAM 50 Bio-amplifier, World Precision Instruments). Depolarisations were amplified ×50, filtered at 0.3 kHz, and recorded onto a chart recorder (Lectromed Multi-Trace 2). Capsaicin (Caps, 1µM, Sigma), Resiniferatoxin (RTX, 3nM, Sigma), Bradykinin (BK, 3 µM, Sigma), Acrolein (Acro, 300µM, Sigma), Prostaglandin-E_2_ (PGE_2_, 10µM, Sigma), or low pH (5) were used to initiate depolarisation of the vagal tissue. The effect of theophylline (Sigma-Aldrich, vehicle 0.1% DMSO in KH) was assessed at specified concentrations. The mechanism of action of theophylline was investigated using the potassium channel blockers paxilline (1µM, Sigma), apamin (1µM, Sigma), glibenclamide (10µM, Sigma) and clotrimazole (10µM, Sigma).

*In vivo staining of airways neurons*

DiI (DilC_18_(3))-(1,1’-dioctacetyl-3,3,3’,3’-tetramethyl-indocarbocyanine perchlorate, Invitrogen) was prepared in 100% ethanol (25mg/ml) and dissolved by ultrasonication overnight, in the dark. On the day of dosing, DiI stock solution was mixed with 0.9% saline (25µl/ml) immediately prior use and instilled intra-nasally using a Gilson^TM^ pipette and a fine tip (1ml/kg). Tissues were collected for analysis 14 days after the procedure.

*Enzymatic Dissociation of Sensory Neurons from Jugular Ganglia*

The jugular ganglia were dissected from the same guinea pigs used for vagus nerve experiments, and separately placed in ice cold Hank’s balanced salt solution (HBSS: in mM KCl 5.33; KH_2_PO_4_ 0.44; NaCl 133.0; Na_2_HPO_4_ 0.3; glucose 10.0; HEPES 5.0; pH 7.4 with NaOH). The dissected ganglia were carefully cleaned of remaining connective tissue under a microscope in a sterile hood. The jugular ganglia were first incubated for 5 min with type IV collagenase (Worthington, USA) in the presence of 300 μM free calcium. Enzymatic digestion was stopped by immersion of the ganglia in ice cold HBSS (5 times the volume of the enzymatic solution). The pre-digested ganglia were then collected with fine tweezers and incubated for 30 minutes in activated Papain (Sigma) 20 U/ml solution at 37°C; the solution being agitated every 5 minutes. The solution was then changed for a Collagenase IV (Worthington, USA) 2 mg/ml and Dispase II (Roche) 2.4 mg/ml containing solution at 37°C. The cells were incubated for 40 min and the solution was gently agitated every 10 min. After 2^nd^ and 3^rd^ enzymatic isolation, tissues were pelleted by centrifugation (410 *g* for 2 min) and the supernatant was carefully removed using sterile disposable plastic Pasteur pipettes. After the 3^rd^ enzymatic isolation, the dissociated ganglia and tissue pellet was triturated in 1 ml room temperature HBSS using F12 medium-coated and fire-polished glass Pasteur pipettes with decreasing diameter from 1 mm to 0.3 mm. The released neurons were collected by centrifugation in a density gradient medium (L15 medium containing 20% Percoll, Sigma) at 25°C. The neuron-containing fraction was homogenised in 2 ml room temperature L15 Medium to wash off the Percoll. The L15 medium was discarded after centrifugation (1100 *g* for 3 min) and the neurons were resuspended in 250 μl complete F-12 medium containing 10% Foetal Bovine Serum (Invitrogen) and 10 µl/ml Penicillin/Streptomycin (Invitrogen, 200 U/ml).

*Primary Culture of Jugular Sensory Neurons*

Calcium imaging of primary sensory jugular ganglia was performed as previously described (E6). The neurons were plated onto culture dishes double coated with 0.01% Poly-L-Lysine (World Precision Instruments) and Laminin (Gibco-Invitrogen, 40 µg/ml) in complete F-12 medium (Invitrogen) containing 10% Foetal bovine serum and 1% penicillin/streptomycin (Invitrogen). Camptothecin (20 µM) (Sigma) was added to the culture medium to inhibit Schwann cells and fibroblasts growth. The neurons were incubated for 24 h (95% O2 and 5% CO2 at 37°C) and used within 10 h afterward. No growth factor was added to the medium in culture.

*Loading of neuron with fluorescent dyes and imaging*

Intracellular calcium and membrane voltage changes were simultaneously measured in primary cultured dissociated jugular neurons. The neurons were loaded with Fura2-AM (5 µM; Invitrogen, 40 min) at room temperature followed by 30min resting to de-esterify the Fura2-AM in the dark. Neurons were then loaded with 40 µM Di-8-ANEPPS (Invitrogen, 20 min) in the dark at room temperature for 20 min. The responsiveness of the cells were assessed with responses to 50 mM potassium solution (K50, in mM: 50 KCl, 101.4 NaCl, 1 MgCl_2_, 2.5 CaCl_2_, 0.33 NaH_2_PO_4_, 10 D-Glucose, 10 HEPES; 297mOsm, pH adjusted to 7.4 with NaOH at 37°C) at the start and end of experiments. Culture petridishes were placed onto the stage of a widefield inverted microscope (Zeiss Axiovert 200) inside a full incubation chamber with temperature and CO_2_ maintained at 37°C and 5% respectively. Cells were excited with a Xenon gas arc lamp’s light driven at 120W (Cairn, ARC Optosource Illuminator) and filtered by 335/7 nm and 387/11 nm filters for Fura-2 and 472/30 nm for Di-8-ANEPPS using filter wheels. Background fluorescence values were obtained by measuring the fluorescence in a neuron free area for each wavelength and were substracted from the neuronal 340, 380 and 472 nm measurements before calculating the 340/380 and F/F0 ratio values. Light emissions were filtered at 520/10 nm for Fura-2 (BS 410) and 660/50 nm for Di-8-ANEPPS (BS 515) and recorded using LD Achroplan 40x 0.60 KORR Ph2 objective and a Hamamatsu EM-CCD C9100-02 camera driven by Simple-PCI software (Hamamatsu). Airway neuron staining with DiI was assessed using 531/40 nm and 593/40 nm excitation/emission filters (BS 565). The cells were constantly perfused with HEPES buffered physiologic extracellular solution (ECS, in mM: 5.4 KCl, 136 NaCl, 1 MgCl_2_, 2.5 CaCl_2_, 0.33 NaH_2_PO_4_, 10 D-Glucose, 10 HEPES; 297 mOsm, pH adjusted to 7.4 with NaOH at 37°C) held at 37°C using a house-designed pressurized perfusion and solution changing system allowing rapid bath volume (0.6 ml) replacement in 3 s. Prior to the experiment the cells were perfused for 5-10 min with ECS to wash off the culture medium. Membrane depolarisation and calcium influx were elicited using capsaicin (1 µM). A concentration response curve to theophylline (in µM: 0.01, 0.1, 1 and 10) was performed in order to determine the concentration which inhibited the capsaicin response by 50% which was later used on airway specific stained neurons. In some experiments, the resting free intracellular calcium concentrations were calculated using the Grynkiewicz method: Ca2+=Kd[(R-Rmin)/(Rmax-R)](Fd/Fs) where Rmin and Rmax are the ratios obtained in absence and in presence of excess calcium, and Fd/Fs the ratio of 380 nm fluorescence in these two conditions (E7). The cells were successively perfused with A-23187 (10µM) in ECS and solutions made of ECS added with various calcium chloride amounts and EDTA (1mM) to achieved free calcium concentrations of 0, 0.1, 0.3, 0.1, 1, 3 and 30 µM. Fluorescence (340 and 380 nm) was recorded until a plateau was reached before perfusing the next solution. In situ Kd was calculated using Kd calculator software available at invitrogen© website ([www.invitrogen.com](http://www.invitrogen.com)) giving a value of 1.081±0.12 µM for primary cultured jugular neurons under our recording conditions.

*Electrophysiology*

Petridishes containing primary cultured neurons were mounted onto a heated stage (TH60-16, MC60, Linkam Scientific Instrument) housed in an Olympus IX-71 inverted microscope and were held at 37°C throughout the experiment. Potassium currents were recorded in a whole cell perforated patch mode and voltage clamp technique using an EPC-9 patch amplifier (HEKA Instruments Inc., New York, USA). Micro-pipettes were pulled from borosilicate glass capillary tubes (O.D. 1.55±0.05 mm, I.D. 1.15±0.05 mm, Hawksley & Sons Limited, Sussex, UK) using a double pull technique on a vertical glass puller (PC-10, Narishige, London, UK) and their tips were fire-polished using a microforge (MF-900, Narishige, London, UK) to achieve a resistance of 2-3 MΩ with the intracellular solution used in this study. Micro-pipettes were advanced to contact the neuron using a PC-6000 Burleigh ® micromanipulator (Thorlabs, Newton, New Jersey, USA) and a high resistance seal (sealing resistance 2-8 GΩ) was formed. The amphotericin-B used to perforate the patched membrane was dissolved in DMSO and diluted in intra-pipette solution to a final concentration of 350 µg/ml (1% DMSO); the Amphotericin B solution was renewed every 2 h. The micro-pipette were front filled with Amphotericin-B free intrapipette solution (in mM: K-Gluconate 120, KCl 20, Choline-Cl 10, HEPES 10 and Sucrose 20. pH 7.2 using KOH, 330 mOsm/L) and back filled using 34 gauge Microfil™ (World Precision Instruments Inc., Saratosa, USA) with an Amphotericin-B containing intra-pipette solution. Pulse ™ software (HEKA Instruments Inc., New York, USA) was used to record and analyse the digitized currents. Signals were filtered at 3 kHz and sampled at 10 kHz. Electrode difference potential was always zeroed before gigasealing. Junction potential was reduced using a 3 M KCl 5% Agar Bridge between recording chamber and the bath electrode immersed in closed chamber containing intrapipette solution. The cells were kept at 37°C throughout experiment with a heated stage insert (World Precision Instrument) and the bath was perfused with warmed solutions (1ml/min) at 37°C. Currents were not leak subtracted. Before recordings, cells were perfused with ECS for 5 min after perforation of the membrane and stabilisation of measured cell capacitance and access resistance. The bath was constantly perfused with ECS at the rate of 1 ml/min which was occasionally changed for modified ECS in which calcium and sodium were substituted with choline (mECS ; in mM: KCl 5.4, Choline-Cl 143.75, MgCl_2_ 1, NaH_2_PO_4_ 0.33, D-Glucose 10, HEPES 10. pH 7.4 using KOH, 321.96 mOsm/L) prior to recordings as stated. All compounds were applied by perfusion (250 µl/min) in direct proximity to the cell. Changes of membrane potential were recorded in zero current clamp mode and elicited by perfusion of theophylline (0.1 µM) or vehicle (0.1% DMSO) containing ECS. For Whole cell perforated patch recordings, potassium currents (*I_K_*) were elicited using 800 ms depolarising square pulses at voltage ranging from -100 mV up to 120 mV (10 mV increments) with a holding potential of -70 mV. Current amplitude was measured as the average of the current value during the last 50 ms of the pulse. Immediately prior to each recording, the ECS was replaced mECS supplemented with tetrodotoxin (300 nM), nifedipine (1 µM), paxilline (1 µM) and glibenclamide (10 µM) to block sodium, calcium and some of the potassium currents apart from those of interest. The perfusion was reverted back to ECS between recordings and for all wash outs. Clotrimazole- and apamin-sensitive currents recorded in control (0.1%DMSO) or theophylline (0.1µM) conditions were calculated by the current difference between control recordings in mECS and currents recorded during perfusion of mECS containing clotrimazole (10 µM) or apamin (1 µM). The blocker sensitive currents were calculated by subtracting the recording obtained in presence of channel blocker from the recording done in control condition before perfusion of the blocker.

For excised inside-out recording, petridishes were mounted as for whole cell patch clamp. Micro-pipettes were pulled from plain capillary tubes (O.D. 1.55±0.05 mm, I.D. 1.15±0.05 mm, Hawksley & Sons Limited, Sussex, UK) using a double pull technique on a vertical glass puller (PC-10, Narishige, London, UK) and their tips were fire-polished using a microforge (MF-900, Narishige, London, UK) to achieve a resistance of 6-8 MΩ. Micro-pipettes were advanced to contact the neuron using a PC-6000 Burleigh micromanipulator (Thorlabs, Newton, New Jersey, USA) and a gigaseal >10 GΩ was formed before the patch membrane was ripped and placed under the nozzle of a fast solution changing perfusion manifold. Currents were recorded in inside-out patch configuration using an EPC-9 patch amplifier (HEKA Instruments Inc., New York, USA). Pulse ™ software (HEKA Instruments Inc., New York, USA) was used to record and analyse the digitized currents. Signals were filtered at 5 kHz using and sampled at 50 kHz. Electrode difference potential was always zeroed before gigasealing. Junction potential was reduced using a 3 M KCl 5% agar bridge between recording chamber and the bath electrode immersed in closed chamber containing intrapipette solution. Inside-out potassium currents were recorded in iso-potassium condition with the intracellular side bath solution composed of (in mM) K-Gluconate 120, KCl 30, HEPES 10 and EGTA 10; pH adjusted to 7.2 using KOH). Bath concentrations of 20 nM and 500 nM of free calcium were achieved by adding 540 µM and 5.9 mM calcium, respectively, to the solutions (osmolarity was adjusted to 330 mOsm/L with 9.46 mM and 4.1 mM sucrose respectively). Channel activity was recorded in control (0.1%DMSO) or theophylline (0.1µM) conditions. Free calcium was calculated using the Stanford University software “Ca-EGTA Calculator v1.3” based on constants from Theo Schoenmakers' Chelator. The micro-pipette was filled with intrapipette solution composed of (in mM; K-Gluconate 120, KCl 30, EGTA 10, HEPES 10 and Sucrose 15; pH adjusted to 7.4 using KOH, 335 mOsm/L). The intra-pipette solution was also supplemented with tetrodotoxin (300 nM), nifedipine (1 µM), clotrimazole (1 µM), paxilline (1 µM) and glibenclamide (10 µM).

To record the potassium currents as close as possible to the physiological conditions the cells were used as soon as their level of attachment to their substrate allowed perfusion, and within 24h of isolation. Membrane depolarisation, which occurs on excitation, opens voltage-gated calcium channels, and the resulting calcium influx modifies the sub-membrane [Ca^2+^]_i_ which in turn increases the activity of the calcium-activated potassium channels SK and IK. We chose a perforated patch configuration to preserve the intracellular content and [Ca^2+^]_i_, but since we wanted to record potassium channel activity as close as possible to the resting state of the neurons prior to any excitation, we replaced the solution surrounding the patched cell with a sodium and calcium free mECS immediately prior and during recording of the potassium channel activity. To prevent calcium store depletion, the perfusion was switched back to ECS between recordings. Clotrimazole (10 µM) - and apamin (1 µM)-sensitive currents were obtained by subtracting the current recorded in presence of the blockers from the current previously obtained in control conditions. A 10 min washout period was then applied before incubation with the theophylline (0.1 µM) and the channel activities were assessed again using the same subtraction method.

*Cigarette smoke exposures:*

Guinea pigs were exposed to cigarette smoke (CS) from research cigarettes (3R4F - with filters removed; University of Kentucky, KY) for 1h, twice daily, with 4h between each bi-daily exposure period. During smoke exposures, food and water was removed, with animals having *ad libitum* access to food and water at all other times. Animals were exposed to cigarette smoke using a negative pressure system; animals were placed into Teague chambers (volume 225L), with a pinch valve connected to one side of the chambers, and an air pump connected to the opposite side. The air pump drew air through the whole system at a constant flow-rate of 1.5L/min. The pinch valve was programmed to alternate between drawing flow through a lit cigarette for 2s, then from the room for 4s. This generated a mock ‘puff’ on the cigarette, with each 6s ‘cycle’ drawing 50ml of smoke followed by 100ml of room air. The air and smoke was drawn into the Teague chamber containing caged but otherwise unrestrained guinea pigs, with a small fan at the bottom ensuring even smoke dispersal. Finally, air was extracted from the opposite side of the chambers.  For air-exposed negative-control comparator groups, the same protocol was applied, except the cigarette was not lit. Each exposure period of one hour consisted of 50 minutes of ‘active’ smoke exposure, with 10 minutes at the end allocated for venting of cigarette smoke. CS exposure protocols were similar to those described in Eltom *et al*., (2012) (E6).

**Compounds and Materials**

*In vivo cough experiments*: Capsaicin (Sigma, UK) was prepared as a 10 mM solution in ethanol and was diluted in vehicle to obtain 1% ethanol and 1% Tween 80 in 0.9% saline. Working solution of citric acid (Sigma-Aldrich, U.K.) was prepared as 0.3 M dissolved in 0.9% saline, (total dosing volume 10 ml/kg i.p). Theophylline was prepared with vehicle (0.5% methylcellulose + 0.2% Tween 80 in saline, 10 ml/kg, i.p., n = 15) at concentration of 0.3, 3, 30, or 100 mg/kg and injected i.p. at a dose of 10 ml/kg one hour prior to exposure to citric acid (0.3 M).

*In vivo single fibre afferents and bronchospasm experiments*: Capsaicin (Sigma-Aldrich, U.K.) was dissolved in 1% ethanol and 1% Tween 80 in 0.9% saline to a working solution of 100 µM. Theophylline (Boehringer Ingleheim) was dissolved in 0.9% saline at a concentration of 100 mg/kg and injected i.v. at a dose of 10 ml/kg 10 min prior to nebulisation of capsaicin. Vecuronium Bromide (Sigma-Aldrich, U.K.) was dissolved in distilled H_2_O at 2.5mg/ml stock and diluted in distilled H_2_O as required.

*In vitro experiments*: Capsaicin, resiniferatoxin, acrolein, theophylline, NS309, UCL1684 and bradykinin were purchased from Sigma-Aldrich (Dorset, UK). PGE_2_ was purchased from Cayman Chemical (Ann Arbor, Michigan, USA). Apamin, glibenclamide, clotrimazole and paxilline were purchased from Tocris (Bristol, UK). Stock solutions were prepared in DMSO except for apamin and bradykinin which were prepared in distilled water, and PGE_2_ which was dissolved in ethanol. On the day of experiment the compounds were diluted in the appropriate medium.

*In vitro Vagus nerve experiments*: Krebs solution was made fresh on a daily basis. All salt were purchased from BDH (Dorset, U.K.). On the day of experiment, stocks were diluted in Krebs to obtain the appropriate concentration with 0.1% DMSO. The low pH solution (pH 5.0) was prepared by titrating HCl into Krebs solution.

*In vitro imaging experiments:* ECS and K50 solutions were made fresh daily. All salts were from B.D.H. (Dorset, U.K.) and Sigma-Aldrich (Dorset, UK). Fura2-AM stocks were prepared in DMSO, frozen at -20°C and diluted in ECS when used. Compounds stock solutions were diluted in ECS to the desired final concentrations. Final vehicle concentration was 0.1%.

*In vitro Electrophysiology experiments:* For patch clamp, ECS, mECS, intrapipette solutions were made fresh on the day of experiment. Amphotericin-B stock solution was prepared in DMSO and diluted in intrapipette solution as needed (0.1% DMSO). For inside-out recording, iso-potassium solutions were made fresh daily. All salts were from B.D.H. (Dorset, U.K.) and Sigma-Aldrich (Dorset, UK). Tetrodotoxin and nifedipine stock solutions were respectively prepared in acidic ECS (pH 5.0) and DMSO and were diluted as appropriate to obtain the working concentration with 0.1% vehicle.

*Data Analysis and Statistics*

Inhibition of capsaicin or citric acid induced cough was analyzed by a non-parametric Kruskal-Wallis test with Dunn's post-hoc test. In the *in vivo* single fibre experiments, inhibition was determined by students paired *t* test, comparing responses (absolute values) after treatment to the control stimulus immediately preceding the treatment. Inhibition of agonist induced vagus nerve depolarisation was analyzed by two-tailed paired t test, comparing responses to agonist (in the same piece of vagus nerve) in the absence and presence of antagonist. Blockade of the theophylline inhibitory effect on vagus nerve depolarisation was analysed by the non-parametric Kruskal-Wallis test with Dunn's post-hoc test. Differences between current-voltage relationships obtained before and after incubation with theophylline were analysed using two-way ANOVA followed by Bonferroni posthoc tests. Channels in a patch were considered to reach an open state (n) using a criterion of the 50% crossing method when the instant current (i) was > (n-½)*I/Σn and < (n+ ½)*I/ Σn where Σn and I are respectively the total number of current levels and the highest current amplitude observed in this patch. The unitary current, defined as zero for the closed state (C), was determined as the mean of the best-fit Gaussian distribution of the amplitude histograms. When more than one channel was present in a patch, the unitary current was calculated as the average of the unitary current determined for each observed level. The open probability (NPO) was calculated as the ratio of the time spent in an open state divided by the total time of the analyzed records. In order to determine the effect of theophylline incubation of channels activity, with each patch being acting as its own control, the percentage of change of NPO between control and theophylline conditions (100*(NPO_control_-NPO_TP_)/NPO_control_) was calculated. Currents were analysed using Clampfit 9.0, Fetchan 8, PSTAT 8 (Molecular Devices, Foster City, CA) and Origin 6 softwares (Microcal Software, Northampton, USA). Data are presented as means ± SEM and statistical significance is denoted as * p < 0.05, ** p<0.1 and *** p<0.01.

**REFERENCES**

E1. Birrell, MA, Belvisi, MG, Grace, M, Sadofsky, L, Faruqi, S, Hele, *et al.,* TRPA1 agonists evoke coughing in guinea pig and human volunteers. Am J Respir Crit Care Med, 2009; 180: 1042-7.

E2. Maher, SA, Birrell, MA, Belvisi MG. Prostaglandin E2 mediates cough via the EP3 receptor: implications for future disease therapy. Am J Respir Crit Care Med, 2009; 180: 923-8.

E3. Adcock JJ, Douglas GJ, Garabette M, Gascoigne M, Beatch G, Walker M, *et al.* [RSD931, a novel anti-tussive agent acting on airway sensory nerves.](http://www.ncbi.nlm.nih.gov/pubmed/12569065) Br J Pharmacol. 2003; 138: 407-16.

E4. Belvisi, MG, Patel, HJ, Freund-Michel, V, Hele, DJ, Crispino, N, Birrell MA. (2008). Inhibitory activity of the novel CB2 receptor agonist, GW833972A, on guinea-pig and human sensory nerve function in the airways. Br J Pharmacol, 2008; 155**:** 547-57.

E5. Birrell, MA, Crispino, N, Hele, DJ, Patel, HJ, Yacoub, MH, Barnes, PJ *et al*., Effect of dopamine receptor agonists on sensory nerve activity: possible therapeutic targets for the treatment of asthma and COPD. Br J Pharmacol, 2002; 136**,** 620-8.

E6. Grace M, Birrell MA, Dubuis E, Maher SA, Belvisi MG. [Transient receptor potential channels mediate the tussive response to prostaglandin E_2_ and bradykinin.](http://www.ncbi.nlm.nih.gov/pubmed/22693178) Thorax. 2012; 67:891-900.

E7. Grynkiewicz G, Poenie M, Tsien RY. A new generation of Ca2+ indicators with greatly improved fluorescence properties. J Biol Chem 1985;260 :3440-3450.
